# Comparing Sodium Redistribution, Cooking Performance, Texture, and Sensory Properties of Guar Gum and Semperfresh Salt-Coated Noodles

**DOI:** 10.1021/acsomega.5c12150

**Published:** 2026-02-20

**Authors:** Shin-Yong Yeoh, Ahmad Syahir Zulkipli, Thuan-Chew Tan, Uthumporn Utra, Hui-Ling Tan, Azhar Mat Easa

**Affiliations:** † Food Technology Division, School of Industrial Technology, 26689 Universiti Sains Malaysia, 11800 Penang, Malaysia; ‡ Earth Material Characterisation Laboratory, Centre for Global Archaeological Research, Universiti Sains Malaysia, 11800 Penang, Malaysia; § Renewable Biomass Transformation Cluster, School of Industrial Technology, Universiti Sains Malaysia, 11800 Penang, Malaysia; ∥ School of Science, Monash University Malaysia, Jalan Lagoon Selatan, 47500 Bandar Sunway, Selangor Darul Ehsan, Malaysia

## Abstract

This study evaluated the effects of novel salt coating strategies on the functional and structural properties of the yellow alkaline noodles. Noodles were coated by immersion in 0.15% guar gum or 5% Semperfresh solutions containing 10% NaCl to produce salt-coated noodles. The coatings enhanced Na retention (129–134%) and promoted controlled sodium redistribution during cooking compared with commercial noodles. Improvements included increased lightness, reduced cooking time, lower cooking loss, and a more compact gluten–starch network that limited starch leaching. Microstructural analysis revealed a denser, honeycomb-like protein matrix encapsulating starch granules, while textural measurements showed higher tensile strength, elasticity, hardness, and springiness, consistent with improved sensory scores. For the first time, Na distribution was visualized using stained NaCl, demonstrating how coating matrices modulate ion mobility and reinforce protein–starch interactions. These findings provide mechanistic insights into how salt coatings affect noodle structure and functionality, offering a practical strategy to enhance quality and support the WHO-recommended reduction in sodium intake in foods.

## Graphical abstract

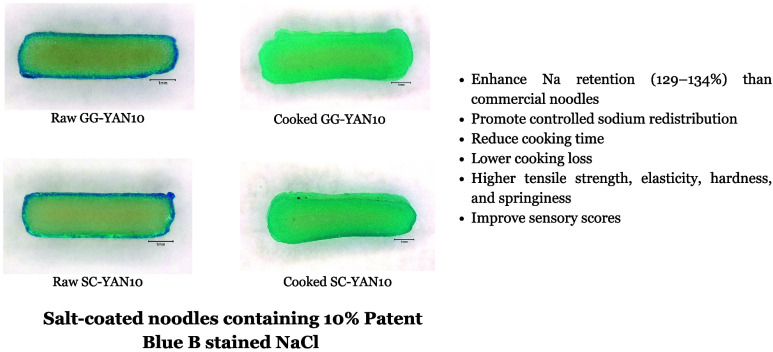

## 1 Introduction

Noodles are a widely consumed cereal-based food with global popularity due to their affordability, convenience, and nutritional value.^1^ Yellow alkaline noodles (YAN), made with *kansui*, a blend of sodium (Na) and potassium carbonates, are distinguished by their characteristic color, texture, and flavor.^2^ With rising living standards, consumers increasingly value noodles for their color, aroma, texture, and enhanced nutritional quality.^3^

Sodium chloride (NaCl), the main component of table salt, is widely used in food processing as a preservative and flavor enhancer.^1^ NaCl is typically added at 0.7–5.0% in noodle production to improve dough elasticity, extensibility, and overall texture.^4,5^ However, excessive dietary Na intake is strongly linked to hypertension, cardiovascular disease, and kidney disorders. Globally, about 100 million adults suffer from hypertension, with 9–17% of cases attributed to high Na consumption,^6^ and excess salt intake contributes to an estimated 5 million deaths annually.^7^ Processed foods account for nearly two-thirds of Na intake in Western diets.^6^ While the World Health Organization (WHO) recommends a daily Na intake below 2 g (5 g salt), global consumption averages 4.3 g Na (≈10.8 g salt), more than double the guideline.^8^ Na reduction is recognized as one of the most cost-effective public health strategies, with the WHO promoting reformulation, front-of-pack labeling, consumer awareness campaigns, and improved food service practices.

Guar gum is a nonionic polysaccharide derived from the endosperm of *Cyamopsis tetragonolobus* (Leguminosae). Recognized as GRAS (Generally Recognized as Safe), it is widely used as a thickening and stabilizing agent, and its high water-binding capacity makes it particularly suitable as an edible coating matrix.^9^ In noodle systems, guar gum has been reported to enhance texture and mouthfeel through interactions with starch and gluten, while also forming surface films capable of retaining solutes such as salt.^10^

Semperfresh is a GRAS-classified edible coating composed of sucrose polyesters, sodium carboxymethylcellulose, and mono- and diglycerides. While it is traditionally applied in fruits and vegetables to control moisture loss and gas exchange, its film-forming ability and hydrophilic–lipophilic balance also make it a promising matrix for salt delivery and retention in cereal-based foods.^11^ Despite their different compositions and conventional applications, both guar gum and Semperfresh can function as salt-carrying coating matrices, providing an opportunity to compare how coating chemistry influences Na retention, redistribution, and subsequent noodle structure and quality.

Approximately 70% of the salt in commercial YAN is lost during cooking, much of it dissolving into soups or sauces without contributing significantly to noodle saltiness.^12^ Salt coatings offer a potential strategy to reduce this loss, enhancing perceived saltiness and functional quality. Previous studies on air-dried YAN demonstrated that coatings with Hylon VII and Semperfresh enhanced Na release into saliva, producing a saltiness perception comparable to conventional noodles.^11,13^ Moreover, noodles with 10% salt coatings exhibited reduced optimum cooking time, stable pH and color, denser structure, improved matrix continuity, and faster salt release, resulting in superior mechanical and textural properties.

The distribution of Na in salt-coated noodles has been examined using guar gum coatings, effectively retaining Na on the noodle surface even after cooking, thereby minimizing leaching losses.^12^ Such retention is vital for preserving Na’s functional role in product quality while contributing to dietary Na reduction. However, the mechanisms by which salt-based edible coatings regulate Na redistribution during cooking and how this redistribution influences gluten–starch structural development and sensory perception in YAN remain insufficiently understood. In addition, indirect visual evidence of Na localization and migration in coated noodle systems is still limited. Moreover, the effects of salt coatings on gluten network organization and starch distribution in salt-coated YAN have not been systematically reported. Semperfresh, an edible coating known for forming protective films, has been applied as a salt-coating matrix for YAN and evaluated for its effects on Na retention, cooking performance, textural properties, and microstructure.^11,13^ This study therefore investigates and compares guar gum and Semperfresh salt coatings, each applied with the same NaCl concentration (10% w/v), to evaluate their effects on Na retention, redistribution, cooking performance, textural and sensory attributes, and microstructure of air-dried YAN under identical Na loading conditions, including visualization of Na localization as well as gluten network organization and starch distribution. It is hypothesized that applying these coatings will enhance Na retention, cooking quality, texture, and sensory acceptability compared with uncoated noodles.

## 2 Materials and Methods

### 2.1 Material

The main ingredients for noodle preparation, including wheat flour, salt, and *kansui* reagent, were sourced from Lotus’s Stores (Malaysia) Sdn. Bhd. (Georgetown, Malaysia). GRINDSTED GUAR 250 guar gum was provided by Danisco Malaysia Sdn. Bhd (Shah Alam, Malaysia). A commercially available YAN sample (COM-YAN), purchased from Sugo Village (Georgetown, Malaysia), was the reference for all analytical evaluations. Additional analytical-grade chemicals were sourced from Sigma-Aldrich (St. Louis, United States). Deionized water was used to conduct all experiments.

### 2.2 Preparation of Fresh YAN

Fresh YAN formulation comprised 100 g of wheat flour (9% protein), 50 g of deionized (DI) water, and 1 g of *kansui* (36% Na_2_CO_3_). It was prepared using the method outlined by Yeoh et al.^12^ The ingredients were mixed by using a Kenwood mixer (London, U.K.). The mixing was started at speed 1 and incrementally increased each min until speed 6, after which it was reduced to speed 1. The dough was then transferred into a plastic bag and sheeted using a Marcato Ampia 150 pasta machine (Campodarsego, Italy). The dough was passed through the roller gap, which was initially set to position 0 (approximately 2.2 mm). The gap was then adjusted to positions 1 (2.0 mm) and 2 (1.8 mm) to achieve the desired thickness. Between each pass, the noodle sheet was folded to ensure an even consistency. A small amount of wheat flour was lightly dusted onto the dough surface during cutting to prevent sticking. The dough sheet was cut into flat, rectangular noodle pieces using the same machine. The noodles were steamed for 30 min in a steamer before being cooled to room temperature. YAN were separated into two categories: guar gum-coated (GG) and Semperfresh-coated (SC).

### 2.3 Preparation of GG-YAN

GG-YAN10 was prepared by dissolving 0.15 g of guar gum and 10 g of NaCl (10% w/v) in deionized water, adjusting the final volume to 100 mL, and immersing fresh YAN in the coating solution for 1 min. This coating method and guar gum concentration were adopted from previous studies.^12^ A control (GG-YAN0) was prepared by using the same process without NaCl. After being coated, the noodles were hung on racks and air-dried at 30 °C for 6 h in a Memmert IN110 incubator (Schwabach, Germany). They were then stored at 4 °C until further analysis.

### 2.4 Preparation of Semperfresh-Coated-YAN

SC-YAN10 was prepared by dissolving 5 mL of Semperfresh and 10 g of NaCl (10% w/v) in deionized water, adjusting the final volume to 100 mL, and immersing fresh YAN in the coating solution for 1 min. This coating procedure and Semperfresh concentration were adopted from previous studies.^11^ A control (SC-YAN0) was prepared using the same method without NaCl. Following coating, the noodles were hung on racks and air-dried at 30 °C for 6 h in a Memmert IN110 incubator (Schwabach, Germany). They were then stored at 4 °C until further analysis.

### 2.5 Determination of Na Content in Noodles by Flame Atomic Absorption Spectrometry (FAAS)

Freeze-dried noodle samples (200–250 mg) were digested using a Multiwave 3000 closed microwave digestion system (Anton Paar, Germany). The absorbance was measured at 589 nm by using a Shimadzu AA-7000 flame atomic absorption spectrophotometer (Japan). Each noodle type was analyzed in triplicate.^13^

### 2.6 Determination of Noodles’ Cooking Properties

Noodle cooking properties were evaluated by determining the optimal cooking time (OCT), cooking yield, and cooking loss.^14^ Noodle samples (15 g) were cooked in deionized water (1:20, w/v). The OCT was determined as the point when the central white core disappeared under compression between two glass plates.

The cooking yield was determined using the equation

$$cooking\;yield\;\left(\%\right)=\frac{weight\;of\;noodles\;after\;cooking}{weight\;of\;noodles\;before\;cooking}\times 100\%$$

Cooking loss was determined by evaporating the cooking water in a hot air oven at 105 °C until a constant weight was achieved. It was expressed as the weight of solid substances leached from the noodles into the cooking water. All measurements were performed in triplicate.

### 2.7 Color Evaluation

The color of cooked noodles was measured using a Minolta Chromameter with a D65 illuminant on the CIE Lab* scale. Triplicate readings were taken at random surface locations.^13^

### 2.8 pH Measurement

The pH of cooked noodles was measured using a Mettler-Toledo Delta 320 pH meter calibrated with pH 4.01, 7.00, and 9.21 buffers. A 10 g sample was homogenized in 100 mL of deionized water for 5 min, allowed to stand for 30 min, filtered, and analyzed. Measurements were performed in triplicate.^13^

### 2.9 Mechanical Properties

Tensile strength and elasticity were measured using a TA-TX2 Texture Analyzer (Stable Micro Systems, Surrey, England) with a Spaghetti/Noodle Tensile Rig and a 5 kg load cell.^13^ The rig was calibrated before analysis. The probe was configured to move apart by approximately 15 mm. The analysis settings were: mode: measure force in tension; option: return to start; pretest speed: 3.0 mm/s; test speed: 3.0 mm/s; post-test speed: 5.0 mm/s; distance: 100 mm. Ten strands of noodles were cooked to their optimal cooking time, drained for 30 s using a sieve, and allowed to cool naturally at room temperature (25 ± 2 °C) for 5 min under ambient conditions. Excess surface water was gently removed by placing the noodle strands on absorbent paper without applying pressure. Tensile measurements were carried out within 10 min after cooking. The width and thickness of each noodle strand were measured at three positions by using a micrometer (Mitutoyo MI 7305, Japan). Tensile strength was calculated as

$$\sigma =\frac{F}{A}$$

where σ denotes the tensile strength (Pa), *F* represents the maximum load or peak force (N), and *A* is the cross-sectional area of the noodle strand (m^2^).

The elasticity modulus was determined as

$$elasticity\;\mathrm{mod}ulus=\frac{Flo}{\mathrm{tA}}\times \frac{1}{v}$$

where *F*/*t* is the initial slope (N/s) of the force vs time graph, *l*_0_ is the original noodles length between limit arms (0.015 m), *A* is the original cross-sectional area (m^2^), and *v* represents the speed of the upper arm (0.003 m/s).

### 2.10 Texture Profile Analysis (TPA)

TPA was conducted using a TA-TX2 Texture Analyzer (Stable Micro Systems, Surrey, England) with a 35 mm cylindrical probe and a 30 kg load cell.^14^ The load cell was calibrated with a return trigger path of 15 mm. Test parameters were set at a speed of 2.0 mm/s, a strain of 75%, and an auto-20 g trigger type, with compression applied during the pretest, test, and post-test phases. Noodle samples were cooked to their optimal cooking times, drained for 30 s, and cooled naturally at room temperature (25 ± 2 °C) for 5 min. Excess surface moisture was removed using absorbent paper before analysis. Three noodle strands were aligned flat on a platform lined with filter paper and secured using double-sided adhesive tape. From the force–time curves, parameters including hardness, springiness, cohesiveness, and chewiness were obtained. Each noodle type was tested in triplicate.

### 2.11 Sensory Evaluation

Sensory evaluation was conducted following the method of Yeoh et al.^13^ with modifications. Ethical approval was obtained from the Human Research Ethics Committee of Universiti Sains Malaysia (JEPeM) (Approval Code: USM/JEPeM/23090697). Written informed consent was collected from all participants. Thirty panelists participated, comprising undergraduate and postgraduate students and staff from the Food Technology department at Universiti Sains Malaysia. Five noodle samples (GG-YAN0, GG-YAN10, SC-YAN0, SC-YAN10, and COM-YAN) were evaluated. Each sample was cooked to its optimal cooking time, allowed to cool naturally to room temperature (25 ± 2 °C), and served in covered paper cups coded with randomly assigned three-digit numbers in a randomized order. Bottled drinking water was provided for palate cleansing between samples. Panelists assessed color, appearance, aroma, taste, chewability, smoothness, springiness, and overall acceptability using a 7-point hedonic scale (1 = strongly dislike, 2 = moderately dislike, 3 = slightly dislike, 4 = neutral, 5 = slightly like, 6 = moderately like, 7 = strongly like).

### 2.12 Indirect Visualization of Na Distribution Using Stained NaCl

The distribution of NaCl in salt-coated noodles was visualized using stained NaCl crystals prepared with the food colorant Patent Blue V.^15^ Briefly, 54 g of NaCl was dissolved in 150 mL of ultrapure water, boiled, filtered into a beaker, and mixed with 0.5 g of Patent Blue V calcium salt. A nylon thread was introduced as a crystallization seed, and the beaker was covered with perforated aluminum foil and stored under vibration-free conditions at room temperature for two months to allow slow crystallization of the stained NaCl, thereby promoting the formation of well-defined crystals suitable for subsequent visualization of Na distribution. After crystallization, the NaCl crystals were dried at 103 °C for 4 h, manually ground using a mortar and pestle, and sieved through a 2 mm mesh to ensure that all particles used for visualization were smaller than 2 mm. For the preparation of salt-coated noodles, the stained NaCl powder was incorporated by dissolving it directly into the guar gum and Semperfresh coating solutions, which were prepared with deionized water, following the procedures described in Sections 2.3 and 2.4, respectively. The resulting stained salt-coating solutions were then applied to fresh YAN using the same coating conditions as those described for GG-YAN10 and SC-YAN10. For comparison, typical fresh YAN was prepared by directly mixing 1 g of stained NaCl into the noodle dough, described in Section 2.2, without applying the coating. All noodle samples were cooked to their OCT, and both raw and cooked noodles were examined using a VHX-7000 Keyence digital microscope (Tokyo, Japan) at 30× magnification.

### 2.13 Scanning Electron Microscope (SEM) Analysis

The microstructure of cooked noodles was examined using an FEI Quanta FEG 650 SEM (Hillsboro) at 150× magnification. Commercial YAN was used as a reference.^11^

### 2.14 Light Microscopy Analysis

Light microscopy was performed to investigate protein–starch interactions in cooked noodles.^12,16^ Noodle samples (∼1 cm) were fixed in 2.5% glutaraldehyde for 4 h, embedded using a Zhejiang Jinhua Kedi Instrumental Equipment KD-BMIV tissue embedding center (Jinhua, China), and sectioned into 10 μm slices with a Leica RM2135 microtome (Wetzlar, Germany). Protein staining was carried out with 2.5% (w/v) Coomassie Brilliant Blue for 1 h, followed by rinsing with 70% methanol. Starch staining was conducted using 50% Lugol’s solution for 1 min, with excess dye removed by rinsing in deionized water. Sections were examined at 300× magnification with a Keyence VHX-7000 digital microscope (Osaka, Japan), and images were captured.

### 2.15 Statistical Analysis

All experiments were performed in triplicate unless stated otherwise. Results are reported as mean ± standard deviation. Statistical significance was determined using one-way analysis of variance (ANOVA) with Tukey’s post hoc test (*p* < 0.05), conducted in SPSS Statistics 26.0 (IBM SPSS Inc., Chicago, IL).

## 3 Results and Discussion

### 3.1 Analysis of Na Content in Noodles

Figure 1 illustrates the Na content and its release during cooking across various noodle types. No significant interaction was observed between the coating type and Na concentration. GG-YAN10 and SC-YAN10 contained higher Na levels, while GG-YAN0 and SC-YAN0 showed lower levels, likely due to *kansui* (1–1.5% for fresh noodles and 0.3–0.5% for steamed varieties), which typically contains Na or potassium carbonates. Commercial YAN (COM-YAN) had the highest Na content (18,393 mg/kg), with a 100 g serving contributing 1,019 mg, exceeding over 50% of the WHO’s 2 g daily limit (WHO, 2023).

**Figure 1:**
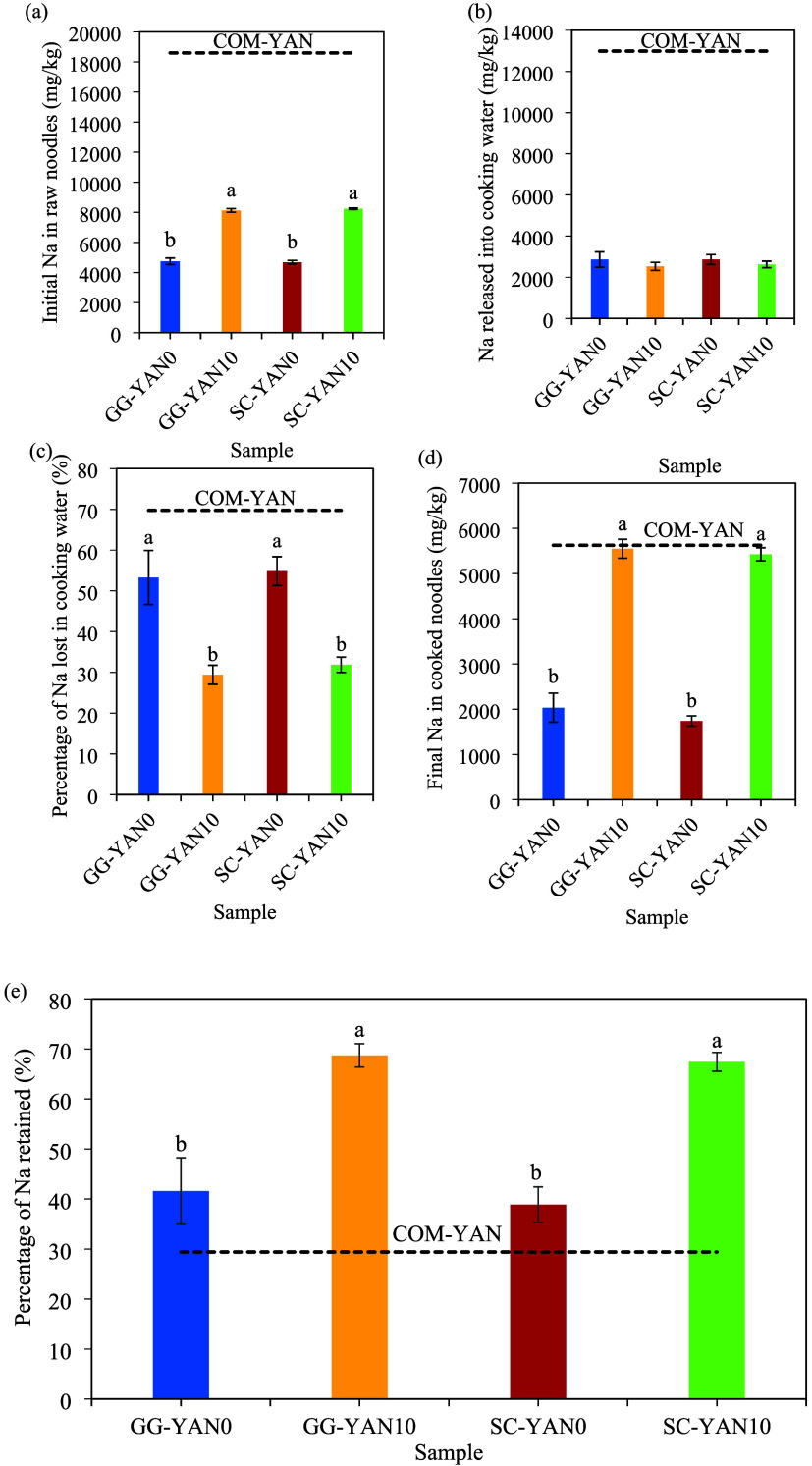
Na content, release, and retention in different types of noodles: (a) initial Na content in raw noodles; (b) Na released into cooking water (mg/kg); (c) percentage of Na lost in cooking water; (d) Final Na content in cooked noodles; (e) percentage of Na retained. Results display mean ± standard deviation (*n* = 3) values. Letters^a,b^ indicate a significant difference (*p* < 0.05) between different bars. COM-YAN was used as a reference and excluded from statistical analysis.

Figure 1b shows no significant differences in Na loss across samples. GG-YAN10 and SC-YAN10 lost 31.3% and 32.6% Na, mainly from the coatings, significantly (*p* < 0.05) less than GG-YAN0 and SC-YAN0, indicating better salt retention (Figure 1c). As a result, cooked GG-YAN10, SC-YAN10, and COM-YAN had similar final Na contents (Figure 1d). Salt enhanced GG viscosity by strengthening intermolecular interactions,^17^ stabilizing the coating, and reducing leaching. Semperfresh, primarily composed of CMC and sucrose esters, showed Na loss comparable to GG. Although NaCl can reduce CMC viscosity,^18^ the sucrose esters and other components likely offset this effect, maintaining coating integrity and salt retention similar to GG. In contrast, COM-YAN released 12,987 mg/kg Na (70.6% loss), likely due to high NaCl concentrations weakening the gluten network, which creates a more open structure that exposes starch. Salt coatings significantly enhanced Na retention in GG-YAN10 and SC-YAN10 compared with COM-YAN, corresponding to a 129–134% increase (*p* < 0.05), while effectively reducing Na leaching during cooking (Figure 1e). All coated noodles outperformed COM-YAN, confirming that salt coatings are an effective strategy to minimize Na loss during cooking while maintaining structural integrity.

### 3.2 Effect on Cooking Qualities

Salt coatings significantly (*p* < 0.05) reduced the OCT of noodles (Figure 2a). High-quality noodles are defined by shorter OCT, higher water absorption, and lower cooking loss.^5^ Noodles with salt coatings exhibited shorter OCT compared with uncoated samples, consistent with previous findings.^13^ This reduction in OCT is attributed to the release of salt during cooking, which enhances water penetration and accelerates starch gelatinization, facilitating gluten network development, and thereby allowing for faster structural softening of the noodle matrix.^19^ COM-YAN had the shortest OCT (2 min) due to parboiling.^20^ During cooking, starch absorbs most of the available water, limiting dough hydration and hindering the development of a strong gluten network.^1^ Salt plays a dual role. At low concentrations, it neutralizes charges on gluten proteins, supporting gradual flour hydration and gluten network development,^12^ while also increasing osmotic pressure, which accelerates water penetration and improves cookability.^21^ OCT has received limited research attention despite its relevance as a cooking quality indicator. The structural integrity of YAN was influenced by coating treatments, which modified the noodle structure in ways that restricted water penetration, regardless of the Na content. This resistance slowed starch gelatinization and ultimately extended the OCT of coated YAN.

**Figure 2:**
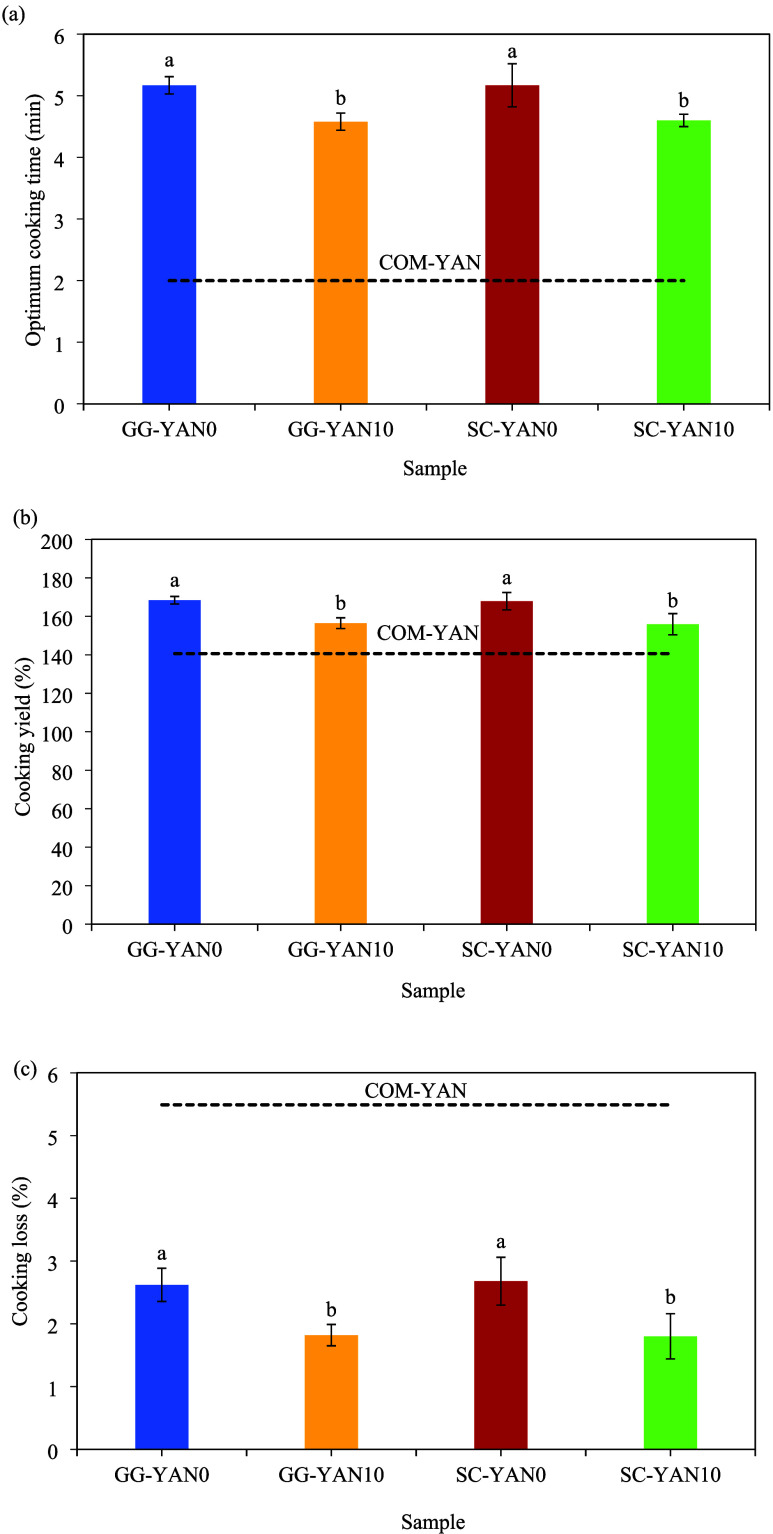
Cooking qualities of noodles. (a) OCT, (b) cooking yield, and (c) cooking loss (%). Results display mean ± standard deviations (*n* = 3) values. Letters^a,b^ indicate a significant difference (*p* < 0.05) between different bars.

Water uptake, measured as noodle weight gain during cooking, is a key quality parameter.^5^ Zero-salt-coated noodles (GG-YAN0 and SC-YAN0) showed significantly higher (*p* < 0.05) cooking yields than salt-coated noodles (GG-YAN10 and SC-YAN10), likely due to their longer OCT, which has been linked to higher yields.^19^ Similarly, a previous study reported reduced water absorption with increasing NaCl levels,^22^ while another observed lower yields with shorter OCT and salt addition,^21^ suggesting that extended cooking times and salt influence hydrophilic interactions, thereby affecting cooking yield.

Cooking loss, a key indicator of noodle integrity, reflects resistance to breakage and disintegration during boiling.^5^ Excessive loss, mainly due to the leaching of salt, starch, and proteins, weakens the protein matrix and clouds the broth.^1,13^ In this study, GG-YAN10 and SC-YAN10 showed significantly lower (*p* < 0.05) cooking losses than GG-YAN0 and SC-YAN0, likely due to the shorter OCT and the presence of salt, aligning with earlier findings.^13^ Moderate salt reduction decreases surface hydrophobicity, strengthens protein interactions, and promotes gluten cross-linking, thereby reducing cooking loss.^23^ By contrast, COM-YAN had the highest loss, as excessive NaCl weakened the gluten and exposed the starch granules.^4^ Notably, all samples in this study had cooking losses below 2.6%, which are well within the acceptable limit of 10% established by the Chinese Agricultural Trade Standards for starch noodles.

### 3.3 Effect on pH

*Kansui* significantly influenced the pH of noodles, while salt coatings had no effect (*p* > 0.05). The sample pH ranged from 7.89 to 8.02,^13^ whereas fresh alkaline noodles made with Na_2_CO_3_ or NaHCO_3_ usually range from 6.5 to 7.0.^5^ Preliminary studies showed that the pH of the GG solution was neutral (pH = 7.0). The elevated pH in this study likely reflects differences in the type and concentration of *kansui*. COM-YAN recorded the highest pH (9.46), suggesting greater *kansui* use, but remained within the Malaysian Standard limit of 10 for wet noodles (MS 2254:2009).^13^

*Kansui* influences both the pH and the protein–starch matrix. Elevating the cooking water pH promotes starch gelatinization and leaching, thereby increasing cooking loss by disrupting amorphous starch regions and hydrogen bonds.^5,24,25^ It also alters the wheat protein composition, increasing albumin and salt-soluble proteins while reducing globulin, gliadin, and glutenin levels.^24^ At higher concentrations, *kansui* increases gliadin solubility, reduces electrostatic repulsion among glutenins, and enhances surface hydrophobicity, resulting in a diminished water-binding capacity and increased starch–protein leaching. These effects were most evident in COM-YAN, which showed reduced cooking yield (Figure 1b) and higher cooking loss (Figure 1c), attributable to destabilization of the protein–starch network.

### 3.4 Effect on Color

High-quality noodles should appear bright and smooth. *Kansui* induces a pH-driven color shift from white to yellow via flavonoid oxidation.^26^ The color attributes of the noodles are shown in Table 1. Salt coatings significantly (*p* < 0.05) increased L* values but did not affect a* or b*. GG-YAN10 and SC-YAN10 showed greater lightness than zero-salt noodles, as salt enhances whiteness.^25^ This increase in whiteness may be attributed to the strengthening of the gluten network by salt, which restricts excessive starch swelling and leaching during cooking, resulting in a smoother and more uniform noodle surface that enhances light scattering. COM-YAN showed lower a* and higher b* values, reflecting its higher *kansui* content and formulation differences. *Kansui* type also influenced color, with Na_2_CO_3_ producing yellow and K_2_CO_3_ green color.^5^ Photographs illustrating the visual appearance of salt-coated noodles before and after cooking are provided in the Supporting Information (Figure S1).

**Table 1:** Color Values for Different Types of Cooked Noodles^a^

sample	L*	a*	b*
GG-YAN0	64.09 ± 1.63^b^	0.37 ± 0.14	21.15 ± 0.39
GG-YAN10	67.41 ± 0.63^a^	0.41 ± 0.07	21.98 ± 0.32
SC-YAN0	64.64 ± 0.73^b^	0.45 ± 0.1	21.39 ± 0.28
SC-YAN10	67.43 ± 0.65^a^	0.35 ± 0.05	21.81 ± 0.32
COM-YAN	67 ± 2.83	–2.36 ± 0.45	32.46 ± 2.92

a Results display mean ± standard deviation (*n* = 3). Letters^a,b^ indicate significant differences (*p* < 0.05) between different columns. *No significant difference (*p* > 0.05) was reported in a* and b* values of cooked noodles. COM-YAN was used as a reference.

### 3.5 Effect on Mechanical and Textural Properties

Noodle texture is a key quality attribute that affects consumer acceptance.^27^ Salt coatings significantly (*p* < 0.05) influenced tensile strength and elasticity, with similar trends observed for both, so only tensile strength is shown (Figure 3a). Salt also significantly affected hardness, springiness, and chewiness but not cohesiveness. Due to similar trends, only hardness data are presented (Figure 3b).

**Figure 3:**
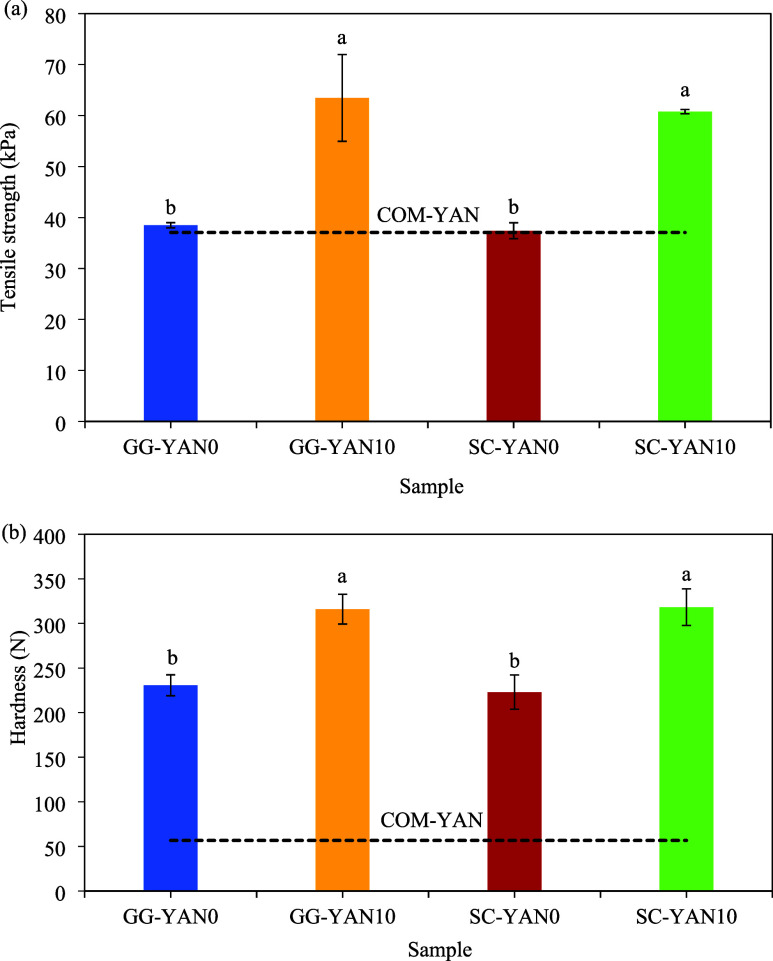
Mechanical and textural properties of cooked noodles: (a) tensile strength and (b) hardness. Results display mean ± standard deviation (*n* = 3) values. Letters^a,b^ indicate a significant difference (*p* < 0.05) between different bars.

GG-YAN10 and SC-YAN10 exhibited higher tensile strength, elasticity, hardness, springiness, and chewiness than zero-salt-coated noodles. The enhancement in hardness and springiness is consistent with previous reports and is attributed to NaCl’s ability to strengthen protein–protein interactions by masking surface charges on gluten proteins, thereby reducing repulsive forces and promoting a stronger gluten network.^25,27^ Salt also improves water–solid, starch–protein, and protein–protein interactions, creating a uniform and compact dough structure.^28^ In contrast, COM-YAN exhibited lower tensile strength and hardness, likely due to excessive Na weakening the gluten network and loosening the noodle structure.^5^

### 3.6 Sensory Evaluation

Table 2 shows the sensory evaluation results. Salt coatings did not significantly affect color, appearance, aroma, or smoothness. COM-YAN scored highest for these attributes, likely due to its yellow color from higher *kansui*, which is consistent with color analysis (Table 1). *Kansui* may also enhance noodle aroma and flavor.^5^ Despite high scores for appearance, COM-YAN received the lowest ratings for hardness and springiness, which align with the texture analysis results (Figure 3). Salt coatings significantly (*p* < 0.05) improved sensory hardness, springiness, and overall acceptability. GG-YAN10 and SC-YAN10 were perceived as firmer and springier than zero-salt-coated noodles, aligning with the mechanical and TPA data (Figure 4). The enhanced texture contributed to higher overall acceptability, indicating a strong correlation between instrumental TPA parameters and sensory perception. Overall, salt coatings positively influenced noodle textural quality and sensory characteristics, highlighting the key role of NaCl in these properties.^5^

**Figure 4:**
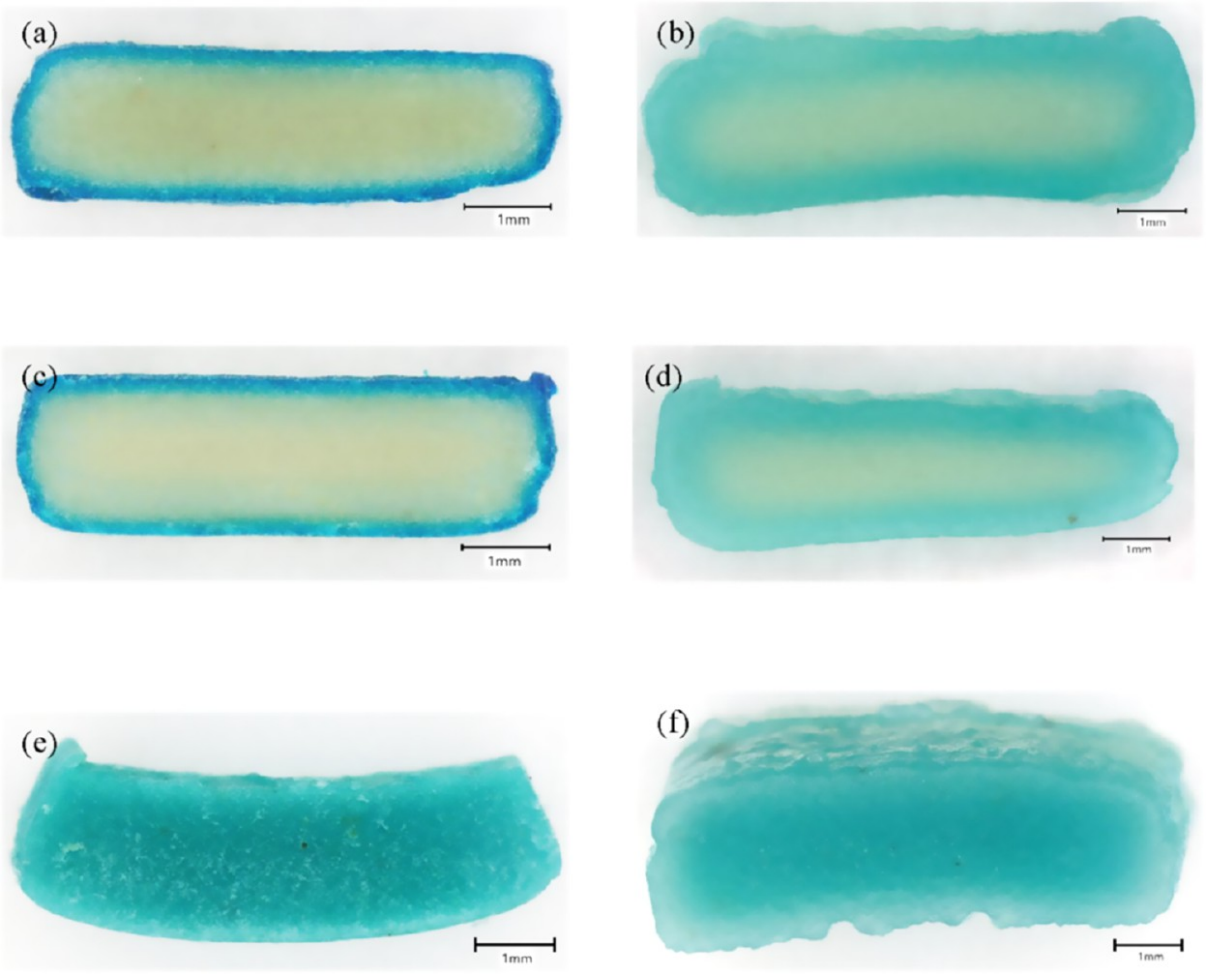
Noodles containing 10% Patent Blue B-stained NaCl. (a) Raw GG-YAN10, (b) cooked GG-YAN10, (c) raw SC-YAN10, (d) cooked SC-YAN10, (e) raw typical YAN, and (f) cooked typical YAN.

**Table 2:** Sensory Evaluation Parameters for Different Types of Noodles^a^

	color*	appearance*	aroma*	hardness	springiness	smoothness*	overall acceptability
GG-YAN0	5.10 ± 1.06	5.13 ± 1.11	4.97 ± 1.25	4.03 ± 1.03^b^	3.90 ± 0.71^b^	4.37 ± 0.85	4.37 ± 1.00^b^
GG-YAN10	4.93 ± 1.08	4.90 ± 1.27	4.93 ± 1.20	5.33 ± 1.09^a^	6.17 ± 0.91^a^	4.33 ± 1.12	5.33 ± 1.24^a^
SC-YAN0	4.97 ± 0.60	5.07 ± 0.63	5.07 ± 0.85	3.87 ± 0.62^b^	3.40 ± 0.66^b^	4.27 ± 0.72	4.03 ± 0.48^b^
SC-YAN10	4.97 ± 0.71	5.07 ± 0.73	5.10 ± 0.83	5.90 ± 0.87^a^	6.07 ± 0.77^a^	4.20 ± 1.01	5.23 ± 0.76^a^
COM-YAN	6.33 ± 0.88	5.50 ± 1.17	5.30 ± 1.37	3.33 ± 0.76	3.40 ± 1.16	5.93 ± 0.87	5.67 ± 1.24

a Results display mean ± standard deviation (*n* = 30). Letters^a‑b^ indicate significant differences (*p* < 0.05) between different columns. *No significant difference (*p* > 0.05) was reported in the color, appearance, aroma, and smoothness of noodles. COM-YAN was used as a reference and excluded from statistical analysis.

### 3.7 Indirect Visualization of Na Distribution Using Stained NaCl

NaCl crystals (<2 mm) stained with Patent Blue V were added to coating solutions to visualize Na distribution. Blue appeared mainly on the raw noodle surface (Figure 4a,c), demonstrating that coatings effectively retained Na externally. After cooking, the blue color faded and spread inward (Figure 4b,d), indicating Na dispersion from the surface to the core due to water penetration, with the noodle core showing lighter blue or yellow, reflecting lower Na concentration.^12^ In contrast, fresh YAN showed uniform blue color throughout raw and cooked noodles (Figure 4e,f), confirming that salt is incorporated directly into the dough and distributes homogeneously during preparation and cooking. The absence of any visible color gradient further supports the migration and solubilization of salt during the kneading and cooking processes.

Unlike earlier salt-coating studies that inferred Na behavior indirectly from sensory or textural responses, the present work provides visual evidence of Na localization and migration using stained NaCl. The Na distribution images clearly demonstrate that salt coatings promote surface Na retention before cooking and regulate inward diffusion during hydration and gelatinization. This controlled redistribution contrasts with the rapid and uniform Na diffusion observed in conventional noodles, highlighting the functional role of coating matrices in Na management. These findings establish a clear mechanistic link between coating-mediated Na localization and subsequent structural and quality outcomes discussed in later sections.

It should be noted that Patent Blue V is a highly water-soluble dye and serves as an indirect tracer of the Na distribution. Although weak, reversible interactions between the dye and the coating solutions cannot be completely ruled out, the observed inward migration and surface fading of the blue color during cooking indicate that the dye predominantly migrated with dissolved Na rather than remaining associated with the coating matrix. Consistent with previous reports, the stained NaCl approach therefore provides qualitative visualization of Na localization and migration, while recognizing that the diffusion behavior of the dye and Na^+^ ions may not be identical.^15^

### 3.8 Effect on Microstructure

During noodle production, gluten proteins form a three-dimensional network that entraps starch granules with cross-linking strengthened by intermolecular bonds during the cooking process. Swelling of starch and interaction with solidified gluten create a dual-network hydrogel with large pores.^3^ GG-YAN0 and SC-YAN0 showed networks with large hollows and voids (Figure 5a,c), indicating underdeveloped gluten due to the absence of salt, which resulted in fragmentation, thinner strands, and structural disruption.^27^

**Figure 5:**
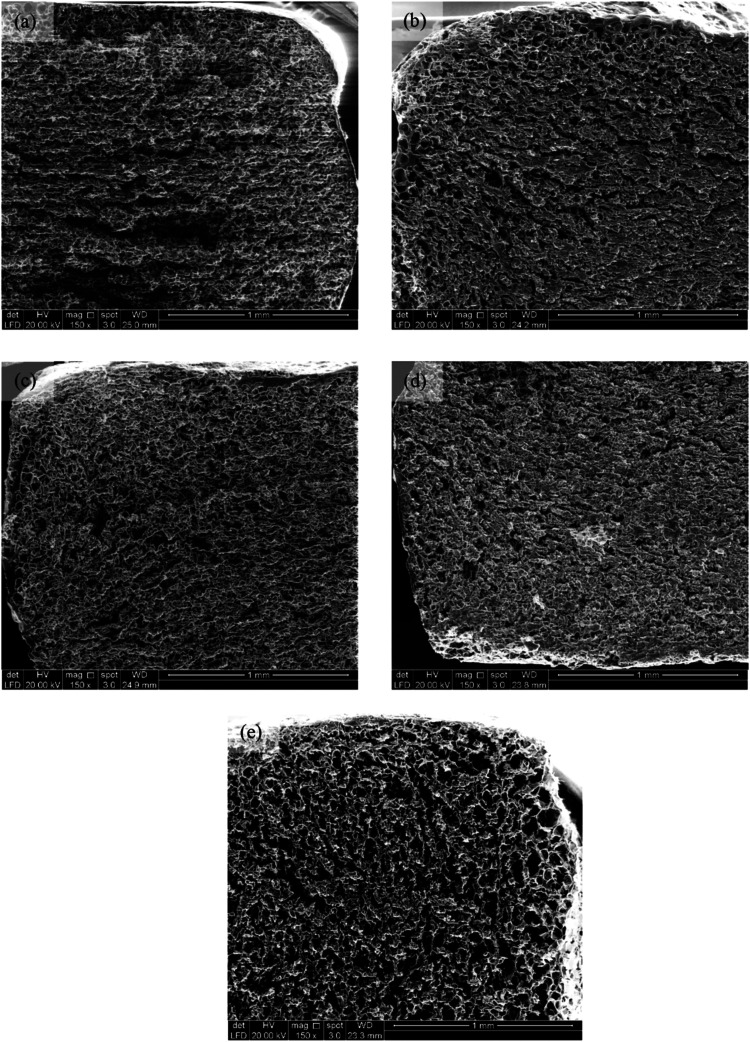
SEM micrographs of the cross-section of cooked noodles at 150× magnification. (a) GG-YAN0, (b) GG-YAN10, (c) SC-YAN0, (d) SC-YAN10, and (e) COM-YAN.

Incorporating 10% salt into GG and SC coatings produced noodles with more continuous, uniform, and honeycomb-like structures (Figure 5b,d). NaCl promotes the formation of fibrous, thread-like gluten structures and strengthens protein interactions, forming a cohesive and resilient network.^25,28^ Salt from the coatings is redistributed during cooking, further enhancing the structural integrity. Dough containing 0–4% salt exhibits a honeycomb-like gluten network with densely packed starch granules, an increased mesh size, and greater resilience compared to unsalted dough.^23,27^ Pores improve water absorption and heat transfer, accelerating starch gelatinization,^29^ while NaCl reinforces gelatinization and overall noodle texture.^25^ This well-developed network and pore combination contributed to shorter OCT and reduced cooking losses in salt-coated noodles (Figure 2).

COM-YAN exhibited a fragmented gluten network with multiple hollows and voids (Figure 6e), indicating a compromised structure. The high initial salt content and direct addition of *kansui* (Figure 1) likely contributed to this disruption, along with parboiling and the shortest OCT (Figure 2a). Excessive salt can hinder cohesive gluten formation by overstrengthening protein interactions, delaying network continuity, and poorly encapsulating starch granules.^4,23^ This weakened structure explains the higher cooking loss observed in COM-YAN (Figure 2c), as the fragmented network allowed for increased starch leaching during cooking.

**Figure 6:**
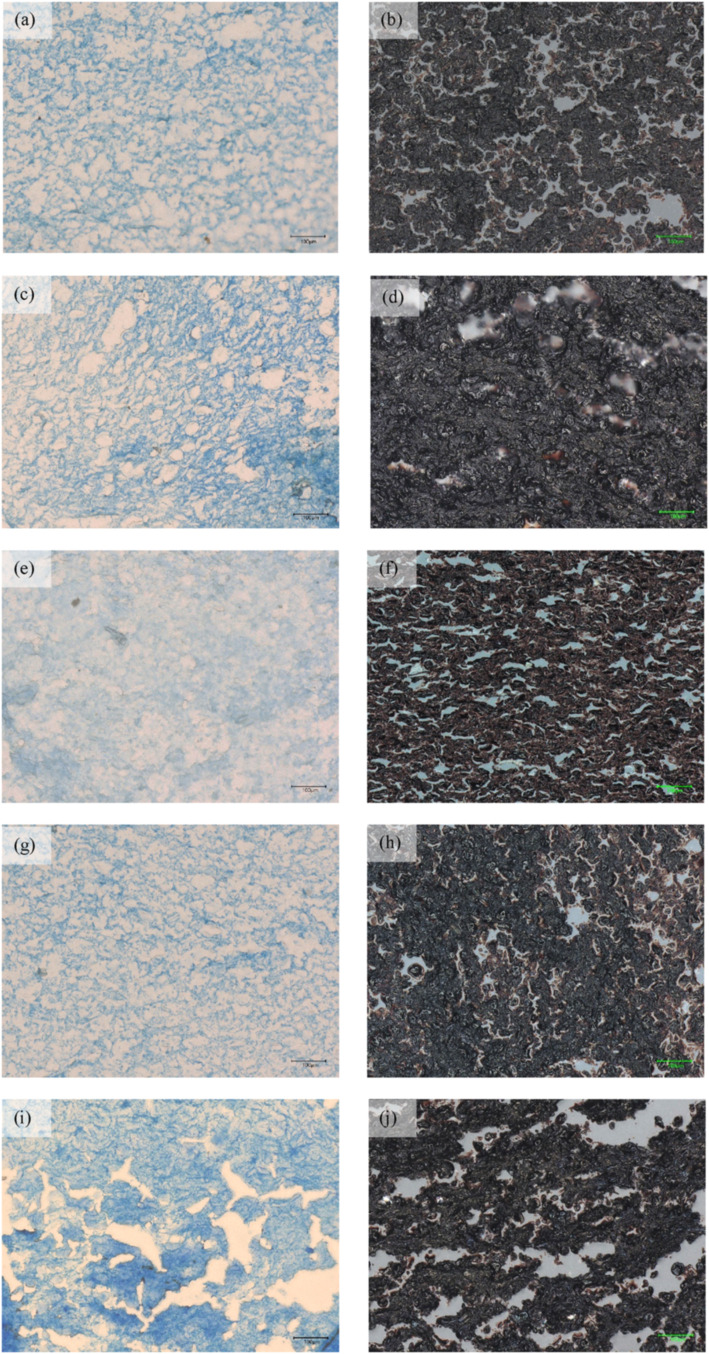
Microstructure of cooked noodles at 300× magnification. (a, b) GG-YAN0, (c, d) GG-YAN10, (e, f) SC-YAN0, (g, h) SC-YAN10, and (i, j) COM-YAN. Proteins are stained blue by Coomassie Brilliant Blue, indicating the gluten network, while starch granules appear dark brown to black following Lugol’s iodine staining.

### 3.9 Effect on Gluten Network and Starch Distribution

Figure 6 illustrates the gluten network and starch distribution in the cooked noodles. GG-YAN0 (Figure 6a) and SC-YAN0 (Figure 6e) showed less dense, irregular gluten networks, with smaller, dispersed starch granules, indicating lower gelatinization, consistent with.^16^ In contrast, GG-YAN10 (Figure 6c) and SC-YAN10 (Figure 6g) exhibited denser, continuous, and well-organized gluten networks. NaCl enhanced noncovalent interactions, promoted β-sheet formation, and developed a fibrous network, resulting in a stronger and more resilient structure.^4,25^ Starch granules in GG-YAN10 (Figure 6f) and SC-YAN10 (Figure 6h) were larger, swollen, and intact, reflecting increased gelatinization. Appropriate NaCl levels strengthen gluten, increase network density, and tightly encapsulate starch granules, thereby improving textural properties in salt-coated noodles.^1,5^

These microstructural observations further demonstrate that Na redistribution is closely linked to gluten network organization and starch distribution. Salt-coated noodles exhibited denser and more continuous gluten matrices, which restricted excessive starch swelling and leaching during cooking. These structural features are consistent with the reduced cooking loss, shorter optimal cooking time, and enhanced tensile and textural properties observed in the salt-coated samples. These results confirm that Na localization is not merely a compositional factor but a key structural driver governing noodle quality through its influence on protein–starch interactions. By applying guar gum and Semperfresh as separate salt-coating matrices under identical Na loading conditions, this study enables a direct comparison of how different coating systems regulate Na retention and redistribution. The observed similarities and differences in gluten network organization and starch distribution between GG- and Semperfresh-coated noodles underscore the role of coating chemistry and film-forming characteristics in modulating Na behavior and associated structure–property relationships.

In COM-YAN (Figure 6i), the gluten network was discontinuous with large holes and starch granules were widely spaced, reflecting poor entrapment. This disrupted network likely caused higher cooking loss and starch leaching, as gaps weaken the structural support.^1^ High NaCl concentrations may have overstrengthened and then disrupted gluten, exposing starch and reducing starch–gluten interactions.^4,23^ Consequently, it could increase amylose release, cause swollen starch granules, and result in a softer, less firm noodle texture.^30^ These changes explain the lower mechanical, textural, and sensory qualities of COM-YAN.

Although this study focused on YAN, the salt-coating strategy demonstrated here is not limited to this product type. Similar mechanisms of Na localization, controlled diffusion during cooking, and reinforcement of starch–protein matrices are also relevant to other cereal-based foods such as wheat pasta and rice noodles. In these systems, surface-applied salt coatings may likewise regulate ion migration, cooking stability, and textural development, suggesting the broader applicability of this approach across diverse cereal matrices.

## 4 Conclusions

Salt coating represents an effective strategy for YAN, enhancing Na retention (129–134% increase compared with commercial noodles) and reducing Na leaching during cooking. This approach improved key physicochemical properties, including increased lightness, reduced optimal cooking time and cooking loss, and reinforcement of the gluten–starch network, thereby limiting structural breakdown. Mechanical and textural attributes, such as tensile strength, elasticity, hardness, and springiness, were enhanced, resulting in improved sensory acceptability. For the first time, Na localization and redistribution in salt-coated noodles were indirectly visualized using stained NaCl, establishing clear structure–property relationships that link Na behavior to gluten network organization, starch distribution, and cooking performance. By a comparison of guar gum and Semperfresh coatings under identical Na loading conditions, this study demonstrates how coating matrices govern Na retention and structural development. Beyond improving the noodle quality, the coating approach aligns with WHO Na-reduction strategies by enabling controlled Na delivery and the incorporation of functional additives. The simple, scalable process is compatible with different noodle types. It provides mechanistic insight into the redistribution of Na and protein–starch interactions during cooking, supporting the practical development of healthier noodle products.

## Supplementary Material



## Data Availability

The data supporting the findings of this study are not publicly available due to their relevance to ongoing and unpublished work but are available from the corresponding author upon reasonable request.

## Notes

Images of SC-YAN10 noodles before and after cooking (Figure S1) (PDF)

## #

Also known by the name Sapina Abdullah.
