# Supplementary material for: Comparing Sodium Redistribution, Cooking Performance, Texture, and Sensory Properties of Guar Gum and Semperfresh Salt-Coated Noodles
Source: ACS Omega. 2026 Feb 20;11(9):15190–200. doi: 10.1021/acsomega.5c12150 (PMC12980421; doi:10.1021/acsomega.5c12150)
Supplement: Supplementary file 1 [file ao5c12150_si_001.pdf]

**Supporting Information**

# Comparing sodium redistribution, cooking performance, texture, and sensory properties of guar gum and Semperfresh salt-coated noodles

Shin-Yong Yeoh[1]*, Ahmad Syahir Zulkipli[2], Thuan-Chew Tan[1,3], Utra Uthumporn[1], Hui-Ling Tan[4], Azhar Mat Easa[1]*

[1]Food Technology Division, School of Industrial Technology, Universiti Sains Malaysia, 11800 Penang, Malaysia.

[2]Earth Material Characterisation Laboratory, Centre for Global Archaeological Research, Universiti Sains Malaysia, 11800 Penang, Malaysia.

[3]Renewable Biomass Transformation Cluster, School of Industrial Technology, Universiti Sains Malaysia, 11800 Penang, Malaysia.

[4]School of Science, Monash University Malaysia, Jalan Lagoon Selatan, 47500 Bandar Sunway Selangor Darul Ehsan, Malaysia.

*Corresponding author: syongyeoh@gmail.com, syyeoh@usm.my (Shin-Yong Yeoh), azhar@usm.my (Azhar Mat Easa).

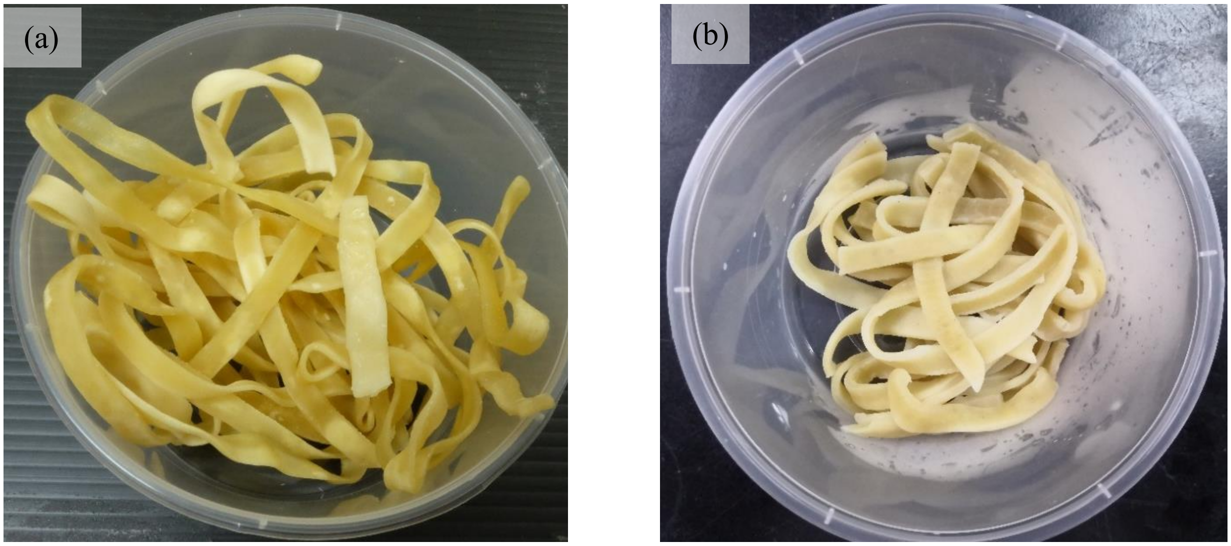

Figure S1. SC-YAN10 noodles. (a) Raw, and (b) Cooked.
